# Supplementary material for: Multiple Novel Human Norovirus Recombinants Identified in Wastewater in Pretoria, South Africa by Next-Generation Sequencing
Source: Viruses. 2022 Dec 7;14(12):2732. doi: 10.3390/v14122732 (PMC9788511; doi:10.3390/v14122732)
Supplement: Supplementary file 1 [file viruses-14-02732-s001.zip › viruses-2038661-supplementary/Table S1 - Final.pdf]

Supplementary Table S1 Norovirus GI and GII viruses and their respective merged reads from sequencing pools

			2018			
**Month**	**P-type**	**Genotype**	**Novel strain**	**Merged reads**	**%**	**Water type**
June	GI.P7	GI.4	Yes	89	0,30	Raw sewage
	GI.P4	GI.4	No	402	1,34	
	GI.P7	GI.7	No	9563	31,97	
	GI.P4	GI.7	Yes	31	0,10	
	GII.P21	GII.6	Yes	13	0,04	
	GII.P7	GII.6	No	19769	66,09	
	GII.P21	GII.13	No	28	0,09	
	GII.P7	GII.13	Yes	17	0,06	
		total		**29912**		
	GII.PNA	GII.4	No	22	8,59	Final effluent
	GII.P7	GII.9	No	234	91,41	
		total		**256**		
July	GI.P13	GI.3	No	318	1,00	Raw sewage
	GI.P7	GI.7	No	13067	40,96	
	GI.P13	GI.7	Yes	7	0,02	
	GII.P16	GII.4	No	12	0,04	
	GII.P7	GII.6	No	12827	40,21	
	GII.P17	GII.6	Yes	531	1,66	
	GII.P7	GII.17	Yes	533	1,67	
	GII.P17	GII.17	No	4606	14,44	
		total		**31901**		
	GII.PNA	GII.4	No	2654	100,00	Final effluent
		total		**2654**		
August	GI.P7	GI.3	Yes	10	0,09	Raw sewage
	GI.P13	GI.3	No	841	7,28	
	GI.P4	GI.4	No	729	6,31	
	GI.P7	GI.4	Yes	11	0,10	
	GI.P13	GI.4	Yes	3	0,03	
	GI.P7	GI.7	No	9945	86,14	
	GI.P13	GI.7	Yes	6	0,05	
		total		**11545**		
	GII.PNA	GII.4	No	1092	100,00	Final effluent
		total		**1092**		
September	GI.P3	GI.3	No	907	4,02	Raw sewage
	GI.P4	GI.3	Yes	132	0,58	
	GI.P7	GI.3	Yes	170	0,75	
	GI.P10	GI.3	No	5	0,02	
	GI.P13	GI.3	No	7	0,03	
	GI.P3	GI.4	Yes	183	0,81	
	GI.P4	GI.4	No	3402	15,07	
	GI.P7	GI.4	Yes	214	0,95	
	GI.P3	GI.7	Yes	99	0,44	
	GI.P4	GI.7	Yes	163	0,72	
	GI.P7	GI.7	No	8769	38,84	
	GII.P7	GII.2	Yes	4	0,02	
	GII.P16	GII.2	No	11	0,05	
	GII.PNA	GII.4	No	4648	20,59	
	GII.P7	GII.4	Yes	57	0,25	
	GII.P7	GII.6	No	13	0,06	
	GII.P7	GII.9	No	3791	16,79	
		total		**22575**		
	GI.P7	GI.7	No	6	1,55	Final effluent
	GII.PNA	GII.4	No	375	97,15	
	GII.PNA	GII.12	No	5	1,30	
		total		**386**		
October	GI.P1	GI.1	No	15279	25,22	Raw sewage
	GI.P3	GI.1	Yes	67	0,11	
	GI.P7	GI.1	Yes	531	0,88	
	GI.P13	GI.1	Yes	276	0,46	
	GI.P1	GI.3	Yes	525	0,87	
	GI.P3	GI.3	No	1480	2,44	
	GI.P7	GI.3	Yes	1317	2,17	
	GI.P13	GI.3	No	3273	5,40	
	GI.P1	GI.7	Yes	658	1,09	
	GI.P3	GI.7	Yes	413	0,68	
	GI.P7	GI.7	No	23442	38,69	

Supplementary Table S1. *Cont.*

2018						
**Month**	**P-type**	**Genotype**	**Novel strain**	**Merged reads**	**%**	**Water type**
October	GI.P10	GI.7	Yes	21	0,03	Raw sewage
	GI.P13	GI.7	Yes	781	1,29	
	GII.P7	GII.2	Yes	49	0,08	
	GII.P16	GII.2	No	3479	5,74	
	GII.PNA	GII.4	No	43	0,07	
	GII.P7	GII.6	No	8894	14,68	
	GII.P16	GII.6	Yes	56	0,09	
		total		**60584**		
	GII.PNA	GII.4	No	662	8,47	Final effluent
	GII.PNA	GII.12	No	6577	84,17	
	GII.P17	GII.12	No	2	0,03	
	GII.PNA	GII.17	No	16	0,20	
	GII.P17	GII.17	No	557	7,13	
		total		**7814**		
November	GI.P3	GI.3	No	14	0,07	Raw sewage
	GI.P7	GI.3	Yes	332	1,65	
	GI.P4	GI.4	No	270	1,34	
	GI.P7	GI.4	Yes	32	0,16	
	GI.P3	GI.7	Yes	13	0,06	
	GI.P4	GI.7	Yes	24	0,12	
	GI.P7	GI.7	No	14135	70,38	
	GII.P7	GII.9	No	5264	26,21	
		total		**20084**		
	GII.PNA	GII.4	No	1972	62,52	Final effluent
	GII.P31	GII.4	No	276	8,75	
	GII.P33	GII.12	No	906	28,73	
		total		**3154**		
December	GII.P7	GII.2	Yes	223	1,61	Raw sewage
	GII.P16	GII.2	No	5103	36,76	
	GII.P7	GII.9	No	8361	60,23	
	GII.P16	GII.9	Yes	194	1,40	
		total		**13881**		
	GII.P16	GII.2	No	1	8,33	Final effluent
	GII.PNA	GII.4	No	4	33,33	
	GII.P7	GII.9	No	7	58,33	
		total		**12**		

2019						
**Month**	**P-type**	**Genotype**	**Novel strain**	**Merged reads**	**%**	**Water type**
January	GI.P4	GI.4	No	617	29,82	Raw sewage
	GII.P16	GII.2	No	547	26,44	
	GII.P7	GII.9	No	439	21,22	
	GII.P16	GII.9	Yes	13	0,63	
	GII.PNA7	GII.12	No	429	20,73	
	GII.P7	GII.12	Yes	6	0,29	
	GII.P16	GII.12	Yes	18	0,87	
		total		**2069**		
	GII.P16	GII.2	No	1478	100,00	Final effluent
		total		**1478**		
February	GI.P7	GI.3	Yes	18	0,93	Raw sewage
	GI.P13	GI.3	No	34	1,75	
	GI.P7	GI.7	No	124	6,39	
	GII.P7	GII.2	Yes	12	0,62	
	GII.P16	GII.2	No	1296	66,74	
	GII.P31	GII.2	No	3	0,15	
	GII.P31	GII.4	No	36	1,85	
	GII.P7	GII.9	No	397	20,44	
	GII.P16	GII.9	Yes	22	1,13	
		total		**1942**		
	GII.P16	GII.2	No	10	100,00	Final effluent
		total		**10**		
March	GI.P3	GI.3	No	10633	23,08	Raw sewage
	GI.P4	GI.3	Yes	28	0,06	
	GI.P3	GI.4	Yes	62	0,13	
	GI.P4	GI.4	No	3311	7,19	

Supplementary Table S1. *Cont.*

2019						
**Month**	**P-type**	**Genotype**	**Novel strain**	**Merged reads**	**%**	**Water type**
March	GII.P7	GII.2	Yes	143	0,31	Raw sewage
	GII.P16	GII.2	No	2073	4,50	
	GII.P7	GII.3	Yes	95	0,21	
	GII.P16	GII.3	No	22	0,05	
	GII.P17	GII.3	Yes	16	0,03	
	GII.P7	GII.6	No	1968	4,27	
	GII.P16	GII.6	Yes	70	0,15	
	GII.P7	GII.9	No	5017	10,89	
	GII.P16	GII.9	Yes	168	0,36	
	GII.P17	GII.9	Yes	73	0,16	
	GII.P7	GII.17	Yes	58	0,13	
	GII.P16	GII.17	No	34	0,07	
	GII.P17	GII.17	No	22299	48,40	
		total		**46070**		
April	GI.P4	GI.4	No	7711	15,23	Raw sewage
	GI.P7	GI.4	Yes	292	0,58	
	GI.P4	GI.7	Yes	330	0,65	
	GI.P7	GI.7	No	10353	20,45	
	GII.P16	GII.2	No	6925	13,68	
	GII.P29	GII.3	No	24602	48,59	
	GII.P16	GII.3	No	420	0,83	
		total		**50633**		
May	GI.P11	GI.6	No	3017	19,30	Raw sewage
	GII.P16	GII.2	No	6604	42,25	
	GII.PNA	GII.2	Yes	137	0,88	
	GII.P16	GII.3	No	137	0,88	
	GII.P29	GII.3	No	5737	36,70	
	total			**15632**		
	GII.P7	GII.6	No	17793	100,00	Final effluent
		total		**17793**		
June	GI.P7	GI.3	Yes	1007	2,37	Raw sewage
	GI.P7	GI.7	No	14429	33,95	
	GI.P8	GI.7	Yes	144	0,34	
	GI.P7	GI.8	Yes	157	0,37	
	GI.P8	GI.8	No	10247	24,11	
	GII.P7	GII.2	Yes	45	0,11	
	GII.P16	GII.2	No	12331	29,01	
	GII.P31	GII.4	No	20	0,05	
	GII.P7	GII.9	No	4080	9,60	
	GII.P16	GII.9	Yes	42	0,10	
	total			**42502**		
	GII.P16	GII.2	No	30800	100,00	Final effluent
		total		**30800**		
July	GI.P7	GI.7	No	4901	15,12	Raw sewage
	GII.P7	GII.2	Yes	71	0,22	
	GII.P16	GII.2	No	4250	13,11	
	GII.P7	GII.3	Yes	20	0,06	
	GII.P29	GII.3	No	1432	4,42	
	GII.P31	GII.4	No	92	0,28	
	GII.P7	GII.7	No	7379	22,77	
	GII.P16	GII.7	Yes	34	0,10	
	GII.P31	GII.7	Yes	8	0,02	
	GII.PNA	GII.9	Yes	32	0,10	
	GII.P7	GII.9	No	14113	43,54	
	GII.P16	GII.9	Yes	80	0,25	
		total		**32412**		
August	GI.P3	GI.3	No	630	1,50	Raw sewage
	GI.P7	GI.3	Yes	338	0,81	
	GI.P3	GI.7	Yes	301	0,72	
	GI.P7	GI.7	No	20346	48,48	
	GII.P7	GII.2	Yes	21	0,05	
	GII.P16	GII.2	No	579	1,38	
	GII.P7	GII.9	No	19737	47,02	
	GII.P16	GII.9	Yes	20	0,05	
		total		**41972**		

Supplementary Table S1. *Cont.*

		2019				
**Month**	**P-type**	**Genotype**	**Novel strain**	**Merged reads**	**%**	**Water type**
August	GI.P7	GI.7	No	4	0,03	Final effluent
	GII.P7	GII.2	Yes	4	0,03	
	GII.P16	GII.2	No	11	0,08	
	GII.P7	GII.9	No	14575	99,87	
		total		**14594**		
September	GI.P8	GI.3	Yes	104	0,25	Raw sewage
	GI.P10	GI.3	No	148	0,35	
	GI.P13	GI.3	No	111	0,26	
	GI.P5	GI.5	No	1252	2,96	
	GI.P8	GI.5	Yes	15	0,04	
	GI.P11	GI.5	Yes	39	0,09	
	GI.P12	GI.5	No	50	0,12	
	GI.P11	GI.6	No	526	1119,15	
	GI.P5	GI.6	Yes	29	61,70	
	GI.P7	GI.7	No	824	310,94	
	GI.P13	GI.7	Yes	10	3,77	
	GI.P8	GI.7	Yes	172	64,91	
	GI.P7	GI.8	Yes	112	0,26	
	GI.P8	GI.8	No	2306	5,45	
	GII.P7	GII.2	Yes	23	0,05	
	GII.P16	GII.2	No	148	0,35	
	GII.P7	GII.3	Yes	203	0,48	
	GII.P8	GII.3	Yes	11	0,03	
	GII.P17	GII.3	Yes	59	0,14	
	GII.P29	GII.3	No	1033	2,44	
	GII.PNA	GII.4	No	165	0,39	
	GII.P7	GII.4	Yes	24	0,06	
	GII.P7	GII.6	No	313	782,50	
	GII.P7	GII.7	No	813	7,60	
	GII.P8	GII.8	No	568	5,31	
	GII.PNA	GII.9	Yes	141	1,32	
	GII.P7	GII.9	No	31294	292,44	
	GII.P8	GII.9	No	70	0,65	
	GII.PNA7	GII.9	Yes	40	0,09	
	GII.P7	GII.16	No	47	0,11	
	GII.PNA7	GII.16	Yes	194	0,46	
	GII.P7	GII.17	Yes	233	0,55	
	GII.P17	GII.17	No	1197	2,83	
		total		**42274**		
October	GII.P7	GII.9	No	10701	38,48	Raw sewage
	GII.P16	GII.9	Yes	265	0,95	
	GII.P7	GII.6	No	181	0,65	
	GII.P7	GII.4	Yes	20	0,07	
	GII.P16	GII.4	No	19	0,07	
	GII.PNA	GII.4	No	171	0,61	
	GII.P16	GII.2	No	5890	21,18	
	GII.P7	GII.2	Yes	222	0,80	
	GII.P16	GII.17	No	53	0,19	
	GII.P17	GII.17	No	1327	4,77	
	GI.P3	GI.3	No	858	3,09	
	GI.P7	GI.3	Yes	338	1,22	
	GI.P11	GI.3	Yes	12	0,04	
	GI.P13	GI.3	No	6262	22,52	
	GI.P5	GI.5	No	8	0,03	
	GI.P11	GI.6	No	297	1,07	
	GI.P13	GI.6	Yes	12	0,04	
	GI.P3	GI.7	Yes	16	0,06	
	GI.P7	GI.7	No	673	2,42	
	GI.P13	GI.7	Yes	421	1,51	
	GI.P13	GI.8	Yes	38	0,14	
	GI.P9	GI.9	No	22	0,08	
		total		**27806**		

Supplementary Table S1. *Cont.*

2019						
**Month**	**P-type**	**Genotype**	**Novel strain**	**Merged reads**	**%**	**Water type**
October	GI.P11	GI.6	No	3	2,94	Final effluent
	GII.P16	GII.2	No	26	25,49	
	GII.PNA	GII.4	No	6	5,88	
	GII.P7	GII.7	No	10	9,80	
	GII.P8	GII.8	No	17	16,67	
	GII.P7	GII.9	No	40	39,22	
		total		**102**		
November	GII.P16	GII.2	No	14	0,04	Raw sewage
	GII.P7	GII.9	No	33312	99,96	
		total		**33326**		

2020						
**Month**	**P-type**	**Genotype**	**Novel strain**	**Merged reads**	**%**	**Water type**
January	GI.P3	GI.3	No	13123	48,47	Raw sewage
	GI.P10	GI.3	No	2472	9,13	
	GI.P13	GI.3	No	11481	42,40	
		total		**27076**		
	GI.P10	GI.3	No	2205	12,58	Final effluent
	GI.P13	GI.3	No	15327	87,42	
		total		**17532**		
February	GI.P1	GI.1	No	190	0,77	Raw sewage
	GI.P13	GI.1	Yes	11	0,04	
	GI.P2	GI.2	No	52	0,21	
	GI.P3	GI.3	No	2225	9,02	
	GI.P4	GI.3	Yes	10	0,04	
	GI.P7	GI.3	Yes	53	0,21	
	GI.P9	GI.3	Yes	16	0,06	
	GI.P10	GI.3	No	666	2,70	
	GI.P11	GI.3	Yes	77	0,31	
	GI.P13	GI.3	No	12870	52,19	
	GI.P4	GI.4	No	400	1,62	
	GI.P11	GI.4	Yes	19	0,08	
	GI.P13	GI.4	Yes	21	0,09	
	GI.P12	GI.5	No	52	0,21	
	GI.P3	GI.6	No	15	0,06	
	GI.P4	GI.6	Yes	7	0,03	
	GI.P6	GI.6	No	63	0,26	
	GI.P11	GI.6	No	3426	13,89	
	GI.P13	GI.6	Yes	159	0,64	
	GI.P7	GI.7	No	279	1,13	
	GI.P13	GI.7	Yes	120	0,49	
	GI.P3	GI.9	Yes	20	0,08	
	GI.P9	GI.9	No	143	0,58	
	GI.P13	GI.9	Yes	16	0,06	
	GII.P16	GII.1	No	13	0,05	
	GII.P17	GII.1	Yes	8	0,03	
	GII.P30	GII.1	Yes	320	1,30	
	GII.P33	GII.1	No	44	0,18	
	GII.P16	GII.2	No	1041	4,22	
	GII.P17	GII.2	Yes	9	0,04	
	GII.P7	GII.6	No	574	2,33	
	GII.P7	GII.9	No	435	1,76	
	GII.PNA7	GII.12	No	14	0,06	
	GII.P7	GII.17	Yes	12	0,05	
	GII.P17	GII.17	No	1269	5,15	
	GII.P33	GII.17	Yes	10	0,04	
		total		**24659**		
	GI.P3	GI.3	No	676	3,26	Final effluent
	GI.P9	GI.3	Yes	28	0,13	
	GI.P10	GI.3	No	608	2,93	
	GI.P13	GI.3	No	17363	83,70	
	GI.P11	GI.6	No	38	0,18	
	GI.P7	GI.7	No	29	0,14	
	GI.P13	GI.7	Yes	27	0,13	

Supplementary Table S1. *Cont.*

2020						
**Month**	**P-type**	**Genotype**	**Novel strain**	**Merged reads**	**%**	**Water type**
February	GI.P9	GI.9	No	339	1,63	Final effluent
	GI.P10	GI.9	Yes	1456	7,02	
	GI.P13	GI.9	Yes	180	0,87	
		total		**20744**		
March	GI.P2	GI.2	No	9	0,05	Raw sewage
	GI.P13	GI.2	Yes	5	0,03	
	GI.P3	GI.3	No	379	2,03	
	GI.P4	GI.3	Yes	4	0,02	
	GI.P6	GI.3	Yes	17	0,09	
	GI.P7	GI.3	Yes	119	0,64	
	GI.P10	GI.3	No	455	2,43	
	GI.P11	GI.3	Yes	14	0,07	
	GI.P13	GI.3	No	8008	42,86	
	GI.P4	GI.4	No	7	0,04	
	GI.P6	GI.6	No	636	3,40	
	GI.P11	GI.6	No	498	2,67	
	GI.P13	GI.6	Yes	38	0,20	
	GI.P7	GI.7	No	751	4,02	
	GI.P13	GI.7	Yes	149	0,80	
	GI.P8	GI.8	No	27	0,14	
	GII.P33	GII.1	No	8	0,04	
	GII.P7	GII.2	Yes	11	0,06	
	GII.P16	GII.2	No	446	2,39	
	GII.P7	GII.6	No	5950	31,84	
	GII.P16	GII.6	Yes	12	0,06	
	GII.P7	GII.7	No	11	0,06	
	GII.P7	GII.9	No	95	0,51	
	GII.P7	GII.17	Yes	6	0,03	
	GII.P17	GII.17	No	1031	5,52	
		total		**18686**		
	GI.P13	GI.3	No	5	0,03	Final effluent
	GII.P33	GII.1	No	11	0,06	
	GII.P7	GII.6	No	17149	99,88	
	GII.P17	GII.17	No	4	0,02	
		total		**17169**		
July	GI.P3	GI.3	No	10	0,04	Raw sewage
	GI.P13	GI.3	No	9377	35,90	
	GI.P7	GI.7	No	55	0,21	
	GII.P7	GII.6	No	16676	63,85	
		total		**26118**		
	GI.P10	GI.3	No	2	6,90	Final effluent
	GI.P13	GI.3	No	3	10,34	
	GII.P7	GII.6	No	20	68,97	
	GII.P7	GII.9	No	2	6,90	
	GII.P16	GII.10	No	2	6,90	
		total		**29**		
August	GI.P10	GI.3	No	6	0,04	Raw sewage
	GI.P13	GI.3	No	6	0,04	
	GII.P7	GII.6	No	53	0,35	
	GII.P16	GII.10	No	15185	99,57	
		total		**15250**		
