# Supplementary material for: Multiple Novel Human Norovirus Recombinants Identified in Wastewater in Pretoria, South Africa by Next-Generation Sequencing
Source: Viruses. 2022 Dec 7;14(12):2732. doi: 10.3390/v14122732 (PMC9788511; doi:10.3390/v14122732)
Supplement: Supplementary file 1 [file viruses-14-02732-s001.zip › viruses-2038661-supplementary/Table S2 - Final.pdf]

Supplementary Table S2. Summary of putative novel GI and GII recombination breakpoints from strains identified in this study.

Study strain	P-type	Genotype	RdRp parental strain	Capsid parental strain	Length	ORF2 starting point	Breakpoint	Breakpoint region
1018RGI.1[P3]	P3	GI.1	MT031988	MK956174	583	270	266	Capsid
1018RGI.1[P7]	P7	GI.1	MH393587	MK956173	579	266	296	Capsid
1018RGI.1[P13]	P13	GI.1	MN922742	MK956175	579	266	329	Capsid
220RGI.1[P13]	P13	GI.1	MN922742	MK956173	579	266	261	RdRp
1018RGI.1[P13]	P13	GI.1	MN922742	MK956173	579	266	263	RdRp
320RGI.2[P13]	P13	GI.2	MN922742	KF306212	579	266	266	Capsid
1018RGI.3[P1]	P1	GI.3	MK956175	MN922742	579	266	262	RdRp
319RGI.3[P4]	P4	GI.3	MK956177	MT031988	579	266	266	Capsid
320RGI.3[P6]	P6	GI.3	MW305503	MT492061	579	266	223	RdRp
320RGI.3[P6]	P6	GI.3	MN421638	MT031988	579	266	266	Capsid
320RGI.3[P6]	P6	GI.3	MW305503	MN922742	579	266	216	RdRp
220RGI.3[P7]	P7	GI.3	MZ298339	MZ022026	579	266	267	Capsid
819RGI.3[P7]	P7	GI.3	MH393584	MT031988	579	266	264	RdRp
320RGI.3[P7]	P7	GI.3	MH393584	MN421673	579	266	270	Capsid
1019RGI.3[P7]	P7	GI.3	MZ298339	MT526277	579	266	269	Capsid
918RGI.3[P7]	P7	GI.3	MH393584	MZ022005	579	266	270	Capsid
320RGI.3[P7]	P7	GI.3	MN453680	MZ022026	579	266	270	Capsid
1019RGI.3[P7]	P7	GI.3	MZ298339	MZ022026	579	266	275	Capsid
919RGI.3[P8]	P8	GI.3	MK762573	MT492067	579	266	273	Capsid
220RGI.3[P9]	P9	GI.3	MT372474	MZ022026	579	266	273	Capsid
220RGI.3[P11]	P11	GI.3	MT357996	MN922742	579	266	275	Capsid
320RGI.3[P11]	P11	GI.3	MT357996	MN922742	579	266	265	Capsid
1019RGI.3[P11]	P11	GI.3	KY210910	MZ022023	579	266	271	Capsid
220RGI.3[P11]	P11	GI.3	MT357996	MT031988	579	266	280	Capsid
320RGI.3[P11]	P11	GI.3	KC294198	MZ021978	579	266	300	Capsid
319RGI.4[P3]	P3	GI.4	MT031988	MT491997	579	266	266	Capsid
918RGI.4[P3]	P3	GI.4	MT031988	MN416765	579	266	289	Capsid
419RGI.4[P7]	P7	GI.4	MH393587	MT491997	579	266	267	Capsid
818RGI.4[P7]	P7	GI.4	JN899243	MT491997	579	266	314	Capsid
918RGI.4[P7]	P7	GI.4	MZ298339	MN416765	579	266	268	Capsid
220RGI.4[P11]	P11	GI.4	MK236611	MT491997	579	266	253	RdRp

Table S2. *Cont.*

Study strain	P-type	Genotype	RdRp parental strain	Capsid parental strain	Length	ORF2 starting point	Breakpoint	Breakpoint region
818RGI.4[P13]	P13	GI.4	MN922742	MT491997	579	266	266	Capsid
919RGI.5[P8]	P8	GI.5	MK762573	MK121731	579	266	228	RdRp
919RGI.5[P11]	P11	GI.5	MT357996	MK121731	579	266	246	RdRp
220RGI.6[P4]	P4	GI.6	MN416765	MN421716	579	266	256	RdRp
919RGI.6[P5]	P5	GI.6	MW305496	MT357996	579	266	241	RdRp
1019RGI.6[P13]	P13	GI.6	MN922742	MT357996	579	266	273	Capsid
220RGI.6[P13]	P13	GI.6	MN922742	KY210910	579	266	271	Capsid
320RGI.6[P13]	P13	GI.6	MN922742	KY210910	579	266	238	RdRp
320RGI.6[P13]	P13	GI.6	MN922742	MN421638	579	266	266	Capsid
1018RGI.7[P1]	P1	GI.7	MK956175	MH393587	579	266	271	Capsid
918RGI.7[P3]	P3	GI.7	MT031988	MH393587	579	266	271	Capsid
918RGI.7[P4]	P4	GI.7	MN416765	MT089580	579	266	329	Capsid
619RGI.7[P8]	P8	GI.7	MK762573	MH393587	579	266	276	Capsid
1018RGI.7[P10]	P10	GI.7	GQ856473	MH393587	579	266	267	Capsid
718RGI.7[P13]	P13	GI.7	MN922742	MH393587	579	266	281	Capsid
619RGI.8[P7]	P7	GI.8	MK762573	MH393587	579	266	274	Capsid
1019RGI.8[P13]	P13	GI.8	MN922742	MK762573	579	266	273	Capsid
220RGI.9[P3]	P3	GI.9	MT031988	MT372474	579	266	230	RdRp
220EGI.9[P10]	P10	GI.9	MW305506	ND	579	266	ND	ND
220RGI.9[P13]	P13	GI.9	MN922742	KX907731	579	266	230	RdRp
220RGII.1[P17]	P17	GII.1	MH638230	KC962459	570	266	266	Capsid
220RGII.1[P30]	P30	GII.1	KF429769	MN461066	570	266	238	RdRp
1218RGII.2[P7]	P7	GII.2	MH251243	MH842207	570	266	279	Capsid
719RGII.2[P7]	P7	GII.2	MH251243	MG893000	570	266	259	RdRp
1019RGII.2[P7]	P7	GII.2	AB233474	MG746004	570	266	254	RdRp
319RGII.2[P7]	P7	GII.2	MW305523	MH842209	570	266	274	Capsid
1018RGII.2[P7]	P7	GII.2	MW305616	MK762634	570	266	274	Capsid
320RGII.2[P7]	P7	GII.2	MW305616	MW305643	570	266	274	Capsid
918RGII.2[P7]	P7	GII.2	MH279830	MG746003	570	266	281	Capsid
219RGII.2[P7]	P7	GII.2	MH251243	MG763371	570	266	281	Capsid
819RGII.2[P7]	P7	GII.2	MH251243	MH842209	570	266	257	RdRp

Table S2. *Cont.*

Study strain	P-type	Genotype	RdRp parental strain	Capsid parental strain	Length	ORF2 starting point	Breakpoint	Breakpoint region
919RGII.2[P7]	P7	GII.2	MH251243	KY441641	570	266	284	Capsid
619RGII.2[P7]	P7	GII.2	MH251243	MG763371	570	266	264	RdRp
220RGII.2[P17]	P17	GII.2	LC486744	MK762634	570	266	264	RdRp
519RGII.2[PNA]	PNA	GII.2	KC962458	LC413797	570	266	276	Capsid
319RGII.3[P7]	P7	GII.3	MH251243	MT678724	570	266	270	Capsid
919RGII.3[P7]	P7	GII.3	MH251243	MH218573	570	266	283	Capsid
719RGII.3[P7]	P7	GII.3	AB233474	MH218717	570	266	253	RdRp
919RGII.3[P8]	P8	GII.3	MZ292794	HM635167	570	266	237	RdRp
919RGII.3[P17]	P17	GII.3	MZ021671	HM635162	570	266	251	RdRp
319RGII.3[P17]	P17	GII.3	MH842230	MT678724	570	266	250	RdRp
918RGII.4[P7]	P7	GII.4	AB233474	MT028542	570	266	238	RdRp
1019RGII.4[P7]	P7	GII.4	MH279830	MK762719	570	266	238	RdRp
919RGII.4[P7]	P7	GII.4	MH279830	MT028542	570	266	308	Capsid
1018RGII.6[P16]	P16	GII.6	MK387050	MH271649	570	266	248	RdRp
319RGII.6[P16]	P16	GII.6	MG745999	MH260511	570	266	304	Capsid
1018RGII.6[P16]	P16	GII.6	MW405900	MH271649	570	266	261	RdRp
718RGII.6[P17]	P17	GII.6	MH842230	MH260511	570	266	267	RdRp
618RGII.6[P21]	P21	GII.6	MN394545	MW661284	570	266	275	Capsid
719RGII.7[P16]	P16	GII.7	MG745998	MH279830	570	266	274	Capsid
719RGII.7[P31]	P31	GII.7	MN308021	MH279830	570	266	252	RdRp
1218RGII.9[P16]	P16	GII.9	MN461122	AY038599	570	266	212	RdRp
219RGII.9[P16]	P16	GII.9	MG745999	AY038599	570	266	288	Capsid
619RGII.9[P16]	P16	GII.9	MG746000	AY038599	570	266	271	Capsid
819RGII.9[P16]	P16	GII.9	MG745998	AY038599	570	266	271	Capsid
319RGII.9[P16]	P16	GII.9	MG746000	AY038599	570	266	248	RdRp
1019RGII.9[P16]	P16	GII.9	MG746000	AY038599	570	266	306	Capsid
119RGII.9[P16]	P16	GII.9	MH842216	AY038599	570	266	277	Capsid
719RGII.9[P16]	P16	GII.9	MG745999	AY038599	570	266	253	RdRp
119RGII.9[P16]	P16	GII.9	MG746000	AY038599	570	266	239	Capsid
319RGII.9[P17]	P17	GII.9	MH842230	AY038599	570	266	287	Capsid
719RGII.9[PNA]	PNA	GII.9	KC962458	AY038599	570	266	298	Capsid

Table S2. *Cont.*

Study strain	P-type	Genotype	RdRp parental strain	Capsid parental strain	Length	ORF2 starting point	Breakpoint	Breakpoint region
919RGII.9[PNA]	PNA	GII.9	KC962458	AY038599	570	266	291	Capsid
919RGII.9[PNA7]	PNA7	GII.9	LC342059	AY038599	570	266	279	Capsid
119RGII.12[P7]	P7	GII.12	AB233474	JQ613569	570	266	280	Capsid
618RGII.13[P7]	P7	GII.13	MN210068	MN394545	570	266	269	Capsid
919RGII.16[PNA7]	PNA7	GII.16	LC342059	AB233474	570	266	276	Capsid
220RGII.17[P7]	P7	GII.17	MW305613	MH842228	570	266	261	RdRp
919RGII.17[P7]	P7	GII.17	MH279830	MH842225	570	266	266	Capsid
718RGII.17[P7]	P7	GII.17	MN210068	MT344182	570	266	247	RdRp
319RGII.17[P7]	P7	GII.17	MN210068	MH842228	570	266	263	RdRp
220RGII.17[P7]	P7	GII.17	MW305616	MZ021956	570	266	243	RdRp
320RGII.17[P7]	P7	GII.17	MW305616	MZ021956	570	266	221	RdRp
919RGII.17[P7]	P7	GII.17	MH279830	MZ021956	570	266	281	Capsid
220RGII.17[P33]	P33	GII.17	KR904231	MH842223	570	266	247	RdRp
